# 

**Published:** 1916-05

**Authors:** 


### Deaths.

Dr. A. J. Fletcher, of Fort Worth, died recently.

Dr. A. H. Boyd, of Forth Worth, died recently.

Dr. Solomon S. Kohn died at his home in Boerne April 7th.

Dr. R. E. Barton, of San Benito, died recently of Bright's disease.

Dr. Alex Irvin died very suddenly at his residence in Wallis March 28th.

Dr. Wilford B. Hardin, a well known young physician of Dallas, died in El Paso April 10th.

Dr. H. M. Markham, who was a practicing physician in Farmersville for a number of years, died in McKinney recently.

Dr. C. H. ("Kit") Williams, a life-long citizen of Matagorda county, died March 30th at the home of Dr. H. L. Rugely, Bay City.

Capt. H. L. Brown, of the medical corps, who was in charge of the ambulance corps with General Pershing's column, died at Fort Bliss hospital, El Paso, April 15th, of heart disease.

